# Lowering the recommended age for the free and active offer of influenza vaccination in Italy: clinical and economic impact analysis in the Liguria region

**DOI:** 10.1080/21645515.2020.1810494

**Published:** 2020-10-29

**Authors:** Cecilia Trucchi, Marco D’Amelio, Daniela Amicizia, Andrea Orsi, Idalba Loiacono, Roberta Tosatto, Maria Francesca Piazza, Chiara Paganino, Andrea Pitrelli, Giancarlo Icardi, Filippo Ansaldi

**Affiliations:** a Planning, Epidemiology and Prevention Unit, A.Li.Sa. Liguria Health Authority, Genoa, Italy; b IRCCS San Martino Hospital, Genoa, Italy; c GSK, Verona, Italy; d Department of Health Sciences, University of Genoa, Genoa, Italy

**Keywords:** Influenza, immunization, prevention, influenza vaccine strategy, lowering influenza vaccine recommendation, influenza clinical impact, influenza economic impact, influenza burden

## Abstract

**Objective:**

we estimated the epidemiological and budget impact of lowering the recommended age for influenza immunization with quadrivalent vaccine actively offered and administered free of charge to persons over 50 years old by public immunization services.

**Methods:**

a multi-cohort, deterministic, static Markov model was populated by real-world data on the clinical and economic impact of Influenza-Like Illness and Lower Respiratory Tract Infection over 1 year. Four scenarios featuring different vaccine coverage rates were compared with the base case; coverage rates in subjects with and without risk factors were considered separately.

**Results:**

compared with the base case, adopting scenarios 1–4 would reduce the annual number of influenza cases by 6.5%, 10.8%, 13.8% and 3.4%, Emergency Department accesses by 10.7%, 9.1%, 15.4% and 4.6%, complications by 8.9%, 9.9%, 14.7% and 4.1%, and the hospitalization of complicated cases by 11%, 9.1%, 15.4% and 4.5%, respectively. The four scenarios would require an additional investment (vaccine purchase and administration) of €316,996, €529,174, €677,539, and €168,633, respectively, in comparison with the base case. Scenario 1 proved to be cost-saving in the 60–64-year age-group. The incremental costs of implementing the other hypothetical scenarios ranged from 2.7% (scenario 4) to 13.2% (scenario 3).

**Conclusions:**

lowering the recommended age for influenza vaccination to 60 years would allow a high proportion of subjects at risk for severe influenza to be reached and would save money.

## PLAIN LANGUAGE SUMMARY

What is the context?

- In most European countries, influenza vaccination recommendations are age-based and thus cover individuals over 50, 60 or 65 years of age. In Italy, the age limit is 65 years.
- Several studies have shown that influenza vaccination in people aged between 50 and 64 years can be cost-effective, or even cost-saving.

What is new and what is the impact?

- We assessed the effect of lowering the age limit of the vaccination recommendation from 65 to 50 years on disease burden and the budget of the healthcare payer in the Liguria region (Italy).
- We found that covering a higher number of subjects at risk for influenza would reduce the number of cases of illness, emergency department accesses, complications and hospitalizations. In addition, there could be significant savings for the payer.

## Introduction

Although seasonal influenza epidemics are often short-lived and usually cause self-limiting illness, they nevertheless constitute a serious public health concern, in that they are a major cause of morbidity, hospitalizations and mortality, and put an acute strain on healthcare resources.^1–6^

Influenza-related mortality and morbidity arise mainly from complications due to underlying health conditions in any age-group.^7–13^ However, as the prevalence of such conditions increases with age, morbidity and mortality are higher in older adults.^10,14^ Furthermore, medical and societal costs contribute to the burden of influenza in different ways according to the age-groups affected.^15^

Seasonal influenza vaccination is an effective means of preventing much of the morbidity, mortality and related costs of the disease among older adults, subjects at high risk^14,16^ and patients with chronic diseases.

Worldwide, age-based immunization programs target various age-groups, such as individuals over 50, 60 or 65 years old. In addition, despite recommendations to immunize all high-risk subjects, vaccination coverage (VC) rates are still low among almost all targeted subgroups in several countries and do not reach the optimal and minimum levels established by the WHO.^17–19^

In 1999, the American Academy of Family Physicians (AAFP) and the US Advisory Committee on Immunization Practices (ACIP) lowered the recommended age limit for routine influenza vaccination from 65 to 50 years.^20^ The rationale behind this decision was that, although many individuals between 50 and 64 years of age were classed as being at risk, only a minority were vaccinated. Today, however, annual vaccination in the US is recommended for all individuals aged 6 months or more.^21^

In December 2009, the European Union (EU) Council adopted a Council Recommendation on seasonal influenza vaccination that encouraged Member States to adopt and implement action plans and policies aimed at reaching seasonal influenza VC of almost 75% among older age-groups and at extending vaccination to people with risk conditions or chronic diseases.^22^ This age-based recommendation was heterogeneously acknowledged by the various EU governments: the majority recommend influenza vaccination for adults aged 65 years and older; in Germany, Greece, Hungary, Iceland, the Netherlands and Portugal, immunization programs include adults aged 60 years and older, while in Malta and Poland, the age limit is 50 years. In Slovenia, influenza vaccination is recommended for all adults aged 18 years and older.^23^

In Italy, influenza vaccination was recommended for subjects at high risk of complications, such as children and adults with high-risk chronic conditions and people aged ≥65 years, subjects who were liable to transmit influenza to the above-mentioned subjects, and some categories of workers.^24^ Specifically, until the 2019–2020 season, influenza vaccination was recommended for elderly persons aged ≥ 65 years, high-risk subjects of all ages and some specific categories, such as healthcare-workers (HCWs). With regard to the next influenza season (2020–2021), when influenza viruses may co-circulate with SARS-CoV-2, the new strategy of the Italian Ministry of Health envisions vaccinating children from 6 months of age and the elderly, including those aged between 60 and 64 years. In addition, influenza vaccination is strongly recommended for HCWs and elderly persons who live in residential or long-term care facilities.^25^

A growing body of evidence indicates that influenza vaccination could also be cost-effective or cost-saving in people aged 50 to 64 years, although the results of these analyzes have been ambiguous and the cost-effectiveness and cost-saving potentials are appreciable only from the societal perspective.^14^ Furthermore, the majority of these analyses were performed before the introduction of the quadrivalent inactivated influenza vaccine (QIV), which has shown greater efficacy and effectiveness than the previous trivalent formulation.^26^ Indeed, studies in adults, adolescents and children >3 years of age demonstrated the non-inferiority in terms of the HI geometric mean antibody titers (GMTs) and seroconversion rates (SCRs) to the common 3 strains compared with licensed trivalent vaccines (TIVs), and demonstrated the superiority in terms of the HI GMT and SCR of the added influenza type B lineage compared with TIVs containing either the B/Yamagata or B/Victoria lineages. Furthermore, modeling studies highlighted the added value of QIVs deriving from its capacity to provide broader immunity against influenza B, thereby reducing the likelihood of a mismatched season.^26^

These improvements, along with the higher costs of vaccination, make it necessary to populate the epidemiological and budget impact models of new immunization strategies with additional parameters. Considering all the above-mentioned issues, evaluating the health and economic effects of lowering the recommended age limit for universal influenza immunization from 65 to 50 years and of using the QIV is important in public health decision-making, in order to improve the current policy.

The objective of the present analysis was to estimate the epidemiological and budget impact of introducing into the national influenza immunization campaign in Italy an age-based recommendation targeting persons aged over 50 years in comparison with the current recommendations, which are limited to adults over 65 years old. The impact of lowering the recommended age threshold to include subjects aged 50–64 years was evaluated according to different scenarios involving different VC rates in populations at risk and not at risk.

## Materials and methods

To estimate the clinical and economic impact of lowering the recommended age for flu vaccination in the Liguria Region, a budget impact analysis (BIA) was performed from the regional healthcare service (RHS) perspective by adapting a previously published model;^27^ only direct costs were considered.

As seasonal influenza and associated healthcare expenditure are quantifiable in the short term, the time horizon was set to one year, corresponding to one influenza season. Real-world data on the clinical and economic impact of influenza-like illness (ILI) and lower respiratory tract infection (LRTI) were used to populate the model, and all the assumptions and data sources were based on in-depth literature evidence.

### Model structure

A multi-cohort, deterministic, static Markov model was applied to a 1-year time horizon, reflecting the trend of annual seasonal influenza. The

impact model was constructed in Microsoft Excel (Microsoft Corporation, Redmond, WA, USA) and simulated the natural history of seasonal influenza.

**Figure 1.**
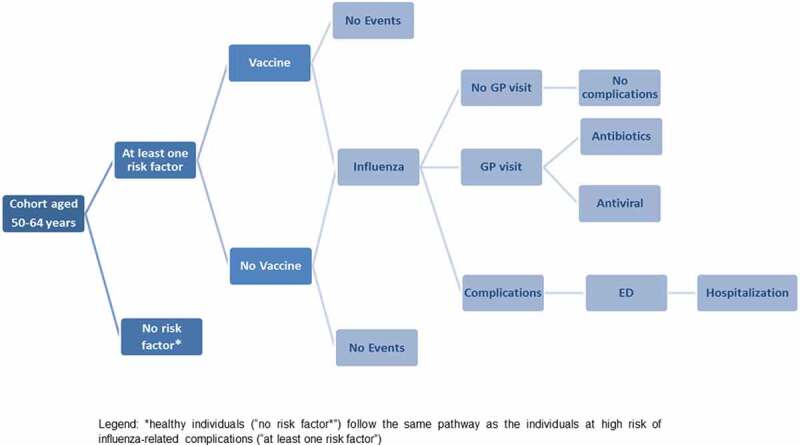
Flow diagram of study population in the model

Briefly, the initial cohort, consisting of the entire Ligurian population aged between 50 and 64 years in 2019, was divided into three age-groups (50–54 years, 55–59 years, 60–64 years) and into two categories: healthy individuals (i.e. without risk factors, NRF) and individuals at high risk of influenza-related complications owing to concomitant diseases (i.e. with at least one risk factor, WRF). These cohorts were entered into the model according to their age and risk category distribution and could receive QIV or not. Each subject could contract influenza or not and, in the former case, could request medical assistance or not and suffer complications or not. Complications could be treated on an outpatient basis or need Emergency Department (ED) access and hospitalization in the most serious cases (Figure 1).

### Population

The resident population in Liguria on 1 January 2019, broken down into the three age-groups of interest, was obtained from the National Institute of Statistics.^28^ The study population was also stratified by risk factors for developing complications with the presence of at least one of the following diseases: renal failure, cancer, diabetes, cardiovascular disease, chronic obstructive pulmonary disease, and gastrointestinal, neurological and autoimmune diseases.^25,29^ The proportions of subjects without risk factors (NRF) and with risk factors (WRF) were calculated by applying the percentages obtained for each of these two populations in the previously published study^30^ (Table 1).

**Table 1.** Input data

	Age group								
	50–54			55–59			60–64		
Input	WRF	NRF	Total	WRF	NRF	Total	WRF	NRF	Total
Target population (n, % [95% CI])	31,158 (23.5% [21.6–24.8])	101,484 (76.5% [75.2–78.4])	132,642 (100%)	34,385 (28.6% [26.8–30.1])	85,927 (71.4% [69.9–73.2])	120,312 (100%)	38,836 (37% [36–37.3%]	66,239 (63% [63–64.1])	105,075 (100%)
Seasonal Incidence of ILI/LRTI ED accesses (per 100 inhabitants), years 2011–2017 (median)	0.483	0.148		0.483	0.186		0.652	0.168	
Probability (%) of ILI/LRTI requiring ED access	7.55	2.31		7.55	2.91		10.19	2.63	
Probability (%) of ILI/LRTI requiring hospitalization among ED accesses (%)			44.4			49.7			62.6
QIV effectiveness *Lineage A* *Lineage B*			61% 73%						
QIV (€)			5.78			5.78			5.78
Cost of administration (€)			6.16			6.16			6.16
ED Access (€)			296.25			296.25			296.25
ED Access followed by hospitalization (€)	2,158	1,416		2,577	1,600		3,134	1,977	
GP consultation *Probability of generating the cost for patients with influenza (%)* *Costs (€)*			60 20.66			60 20.66			60 20.66
Antibiotic therapy *Probability of generating the cost for patients with influenza (%)* *Costs (€)*			47.3 3.06			47.3 3.06			47.3 3.06
Antiviral therapy *Probability of generating the cost for patients with influenza (%)* *Costs (€)*			0.17 38.5			0.17 38.5			0.17 38.5

***Abbreviations. CI: Confidence Interval; WRF: With Risk Factors; NRF: No Risk Factors; ILI: Influenza-Like Illness; LRTI: Lower Respiratory Tract Infection; ED: Emergency department; QIV: Quadrivalent Influenza Vaccine; GP: General Practitioner.***

### Epidemiology of influenza in Italy

Epidemiological data on the last six influenza seasons were extracted from the annual reports by InfluNet, a national network of sentinel General Practitioners (GPs) and Pediatricians (PLS) who report cases of ILI among their patients;^31^ these data are used to estimate the weekly incidence of influenza syndrome during the winter season, in order to describe the duration and intensity of the epidemic (Table 1).

### Emergency Department (ED) Access and Hospitalization

The real-world seasonal incidence rates (per 100 person-years) of ED accesses for ILI and LRTI were obtained from the study published by Trucchi C et al.^30^ Those data were registered in the Genoa Metropolitan Area (GMA) through the syndromic surveillance system (SSS),^32,33^ stratified by risk factors and age-group, and applied to the whole Ligurian population (Table 1). The probability of ED access for ILI/LRTI was calculated from the incidence of ED accesses for ILI/LRTI and the incidence of ILI (6.4%) (Table 1); the percentage of hospitalizations among ED patients was estimated through GMA real-life data, and is reported in Table 1.

### Costs

Table 1 reports the direct costs included in the analysis and the probabilities that subjects with influenza will generate these costs. The cost borne by the regional healthcare system for the purchase of one dose of QIV in the 2018/2019 flu season was set at €5.78. Regarding the costs of administering the vaccine, about 60% of the older adults vaccinated in the Liguria Region are vaccinated by GPs, who receive a fee of €6.16 for this service;^34^ this proportion was applied to the study population.

The cost of ED access was obtained from the Ministerial “Progetto Mattoni”,^35^ actualized to 2017 by a recent Health Technology Assessment analysis, and amounted to €296.25.^36^ The cost of ED access followed by hospitalization due to ILI was calculated through the Diagnosis-Related Group (DRG) system based on the regional system of reimbursement by evaluating the cases registered by the routine data-flows in Liguria.

The analysis also took into account the frequency and the cost of influenza patients with complications.^27^

### Scenarios

In order to address the issue of uncertainty, different scenarios were considered; in line with the regional recommendations for the 2018/2019 flu season, QIV was used in all scenarios. In these scenarios, we distinguished between WRF and NRF subjects because VC is expected to differ according to risk status between the current risk-based strategy and the hypothesized age-based strategy. Specifically, we assumed an increasing level of VC up to a maximum of 50% in the WRF population, which corresponds to the Italian VC achieved in real practice (53.1% in 2019) in subjects aged ≥65 years,^37^ and up to 10% in the NRF population, which corresponds to the Italian VC achieved in real practice in subjects aged 45–64 years (Table 2).^37^

**Table 2.** Base-case and hypothetical scenarios

Scenario	Age-range (years)	Condition	Vaccine coverage (%)	Subjects (n)
Base case	50–64	WRF	26.7%	27,869
		NRF	6.4%	16,233
Scenario 1	50–64	WRF	50%	52,189
		NRF	10%	25,365
Scenario 2	50–64	WRF	35%	36,532
		NRF	25%	63,413
Scenario 3	50–64	WRF	50%	52,189
		NRF	25%	63,413
Scenario 4	50–64	WRF	35%	36,532
		NRF	10%	25,365

*Abbreviations. WRF: With Risk Factors; NRF: No Risk Factors.*

### Base-case scenario

The flu VC rate among subjects aged 18–64 years, with/without at least one chronic disease, was obtained as an aggregate value from the Passi study.^38^ We also used the VC recorded in the general population aged 45–64 years in order to estimate VC in the at-risk population in the same age-range; this latter value was obtained by applying the same proportion between VC in subjects aged 45–64 years and 18–64 years in the general population (Supplementary Table 1).^37^ Estimated VC values of 26.7% and 6.4% were thus obtained for 45–64-year-old WRF and NRF subjects, respectively, and were used in the base-case scenario.

### Other model inputs

QIV effectiveness against type A and type B influenza was assumed to be 61% and 73%, respectively, as estimated by Uhart et al. (2016), and as reported in the meta-analysis by Tricco (2013), respectively.^39,40^

## Results

### Health outcomes

The upper part of Table 3 reports the results of the base-case analysis with regard to the most serious clinical events, i.e. community-acquired influenza diagnosed by sentinel GPs, the proportion of cases requiring ED access, cases with complications and complicated cases requiring hospitalization. The base-case scenario considered the current VC rates of 26.7% and 6.4% in WRF and NRF subjects, respectively, and a regional population of 44,102 vaccinated subjects aged from 50 to 64 years, 63.2% of whom WRF. The model estimated 21,113 cases of influenza, including 7,059 complicated cases and 877 cases (4.2% of all influenza cases) requiring ED access; 52.7% of these latter patients were hospitalized.

**Table 3.** Base-case scenario: health outcomes and costs stratified by age-group and risk factor

Variables (n)	Age-group (years)	WRF	NRF	Total
**Subjects**	50–54	8,319	6,495	14,814
	55–59	9,181	5,499	14,680
	60–64	10,369	4,239	14,608
	**Total**	**27,869**	**16,233**	**44,102**
**Overall cases**	50–54	1,655	6,230	7,885
	55–59	1,826	5,274	7,100
	60–64	2,062	4,066	6,128
	**Total**	**5,543**	**15,570**	**21,113**
**ED access**	50–54	125	144	269
	55–59	138	153	291
	60–64	210	107	317
	**Total**	**473**	**404**	**877**
**Cases with complications**	50–54	886	1,637	2,523
	55–59	978	1,385	2,363
	60–64	1,105	1,068	2,173
	**Total**	**2,969**	**4,090**	**7,059**
**Hospitalization**	50–54	55	64	119
	55–59	68	76	145
	60–64	132	67	198
	**Total**	**255**	**207**	**462**
**Vaccine costs (€)**	50–54	48,084	37,541	85,625
	55–59	53,066	31,784	84,850
	60–64	59,933	24,501	84,434
	**Total**	**161,083**	**93,827**	**254,910**
**Flu shot administration costs (€)**	50–54	30,747	24,006	54,753
	55–59	33,933	20,324	54,257
	60–64	38,324	15,667	53,991
	**Total**	**103,004**	**59,997**	**163,001**
**Clinical costs (€)**	50–54	246,382	360,274	606,656
	55–59	316,320	359,501	675,821
	60–64	586,154	311,884	898,038
	**Total**	**1,148,856**	**1,031,659**	**2,180,515**
**Total costs (€)**	50–54	325,212	421,821	747,033
	55–59	403,319	411,610	814,929
	60–64	684,411	352,053	1,036,464
	**Total**	**1,412,943**	**1,185,483**	**2,598,426**

*Abbreviations. WRF: With Risk Factors; NRF: No Risk Factors; ED: Emergency Department.*

**Figure 2.**
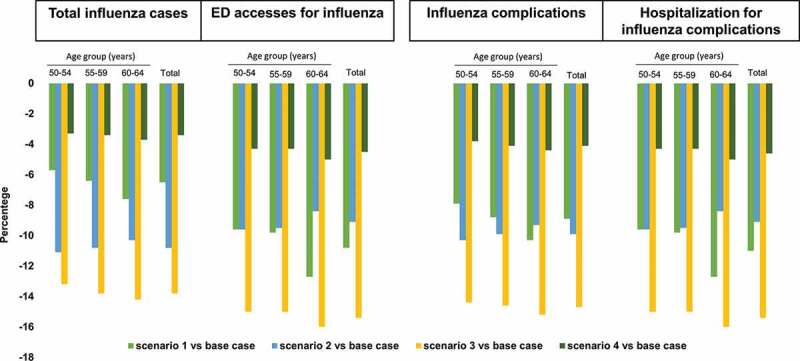
Epidemiological variations between the scenarios and the base case

Figure 2 shows the epidemiological effect of switching to the alternative scenarios 1–4. In comparison with the base case, switching to scenarios 1–4 would reduce the annual number of influenza cases by 6.5%, 10.8%, 13.8% and 3.4%; cases requiring ED access by 10.8%, 9.1%, 15.4% and 4.55%; complications by 8.9%, 9.9%, 14.7% and 4.1%, and hospitalizations of complicated cases by 11%, 9.1%, 15.4% and 4.5%, respectively. The proportions of health outcome measures avoided increase with age in scenarios 1, 3 and 4, while a small age-related reduction is observed in scenario 2. Specifically, in scenario 1, the 60–64 age-group is the cohort with the greatest reduction in the number of total influenza cases (n = 466), influenza cases requiring ED access (n = 40), influenza-related complications (n = 224) and complications requiring hospitalizations (n = 25); these reductions are due to the increase in VC.

### Costs

The *lower* part of Table 3 reports the results of the base-case analysis with regard to the main costs, i.e. vaccination, influenza treatment (including outpatient visits, antivirals and antibiotics), ED accesses, and complications, on distinguishing between outpatient visits and hospitalizations. Each cost category was stratified by age-group and risk factor (Supplementary Table 2).

**Figure 3.**
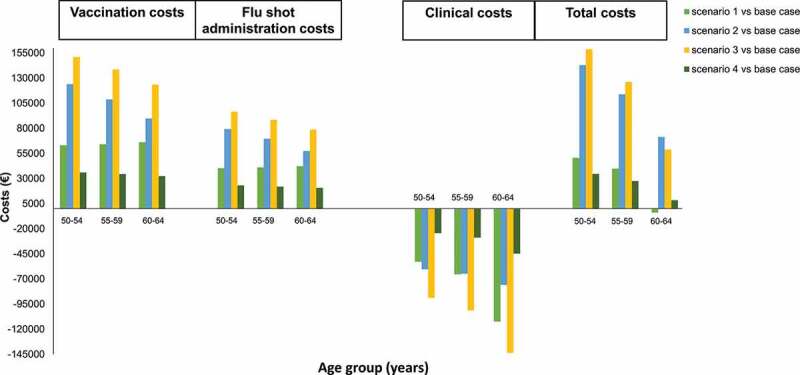
Economic variations between the scenarios and the base case

The base-case scenario envisions vaccine costs of €254,910, flu shot administration costs of €163,000 and clinical costs of €2,180,515 (Table 3). Figure 3 shows the estimated differences in costs between the base case and each of the scenarios hypothesized.

From a budgeting perspective, the scenarios evaluated required a higher financial investment than the base-case scenario; specifically, the estimated additional investment required in scenarios 2 and 3 was almost twice that of scenarios 1 and 4 (scenario 2: €322,778 and scenario 3: €413,276, vs scenario 1: €193,359 and scenario 4: €102,861) (Supplementary Table 2).

On the other hand, in some cases, the total savings resulting from the lower influenza-related clinical costs completely offset the increase in vaccination costs (vaccine purchase and administration). This is the case of the population aged between 60 and 64 years in scenario 1. Indeed, this alternative strategy would allow a saving of approximately € 4,019. (Figure 3). The estimated incremental costs in the other hypothetical scenarios ranged from 2.7% to 13.2% (scenarios 4 and 3 vs base-case scenario, considering the entire population 50–64 years).

## Discussion

The ability of influenza vaccination to prevent illness and to reduce influenza-related hospitalizations and deaths is well known. However, the implementation of immunization policies may require considerable economic investment from the point of view of stakeholders in public health. As economic resources are limited in many healthcare systems, decisions regarding their optimal allocation must be based on solid evidence.

The cost-effectiveness of influenza vaccination has been comprehensively investigated; research has mostly focused on subjects aged 65 years and older, and has indicated that vaccinating older adults is either cost-saving or highly cost-effective.^41–44^ With regard to adults below 65 years old, however, cost-effectiveness studies have yielded heterogeneous results; some have indicated cost savings,^45–47^ while others have not, though they have demonstrated cost-effectiveness.^16,44,48–52^ Furthermore, several studies have been conducted in healthy workers, and their economic evaluations have included the loss of working days.^50,53^

The national immunization plans of some European countries, including Austria, Malta, Slovenia and Poland, recommend vaccination for adults aged 50 to 64 years. In the US, the ACIP has supported this policy since 1999, subsequently extending the recommendation to all subjects aged ≥6 months.^20,21,23,54^ In Italy, since 2012, the main scientific associations of public health specialists, pediatricians and general practitioners have supported the implementation of an age-based strategy, rather than a risk-based strategy, for adults ≥50 years-old.^55^

We assessed the epidemiological and budget impact of a new immunization policy targeting all subjects aged 50–64 years from the perspective of the third-party payer, in comparison with the current strategy, which recommends influenza vaccination only for high-risk individuals in this age-group. The study population, i.e. adults aged 50 to 64 years, includes a large proportion of subjects at high risk of developing influenza-related complications, as well as a large proportion of workers. Specifically, we estimated the impact of progressively increasing VC beyond current levels in terms of the potential avoidance of influenza cases, complications, ED accesses, hospitalizations and costs. The findings of this static model study show that vaccinating 50% of WRF subjects and 10% of NRF subjects aged 60–64 years with QIV can be expected to prevent 466 influenza cases and lower overall healthcare costs by €4,019, in comparison with the current policy. Of note, 37% of subjects aged 60–64 years had risk factors for influenza-related complications.

This study adopted a pragmatic view of the VC likely to be achieved under the new hypothesized strategy. Since it seems unlikely that all subjects aged more than 50 years would be immunized, we assumed that VC would be equivalent to the level achieved in elderly subjects in Italy who are eligible for vaccination under the current policy (about 52.7% in the 2017/2018 influenza season).^56^ Furthermore, the dissection into four different scenarios gives a wide perspective. Specifically, the main advantage of scenario 1 has as is the perspective of reaching the 50% of VC in of subjects WRF, who often are attentive and more involved in their own health care, at the contrary the main disadvantage regards a more high need of healthcare professionals for the realization of the flu campaign. The VC hypothesized in scenario 2 for WRF subjects is easy to reach because it almost corresponds to current obtained VC; the objective fixed for NRF subjects, instead, is more ambitious than the one currently gained but it could allow foreseeing the progressive alignment to an age-based strategy. Scenario 3 targets the vaccination of the higher number of individuals (globally 115,602 subjects) with a high use of public health resources; at the same time, it foresees a more equitable distribution of WRF and NRF subjects to reach. Indeed, this scenario would have the potential to reach a high number of individuals including those who do not know they have a high-risk condition. On the other hand, scenario 4 would involve only 61,897 subjects with a consequent marked easily implementation, from both the low investment of resources and the high feasibility.

As regards the considered age-groups, we found not negligible differences among those investigated (50–54, 55–59 and 60–64 years), with substantial health outcome measures avoided with the increasing of age in scenarios 1, 3 and 4. Particularly, the greatest reduction in terms of number of influenza cases, influenza cases requiring ED access, influenza-related complications and complications requiring hospitalizations was observed in the cohort of 60–64 years and these findings are due to the increase in VC. The advantage of identifying small and homogeneous age-groups is the possibility to estimate the economic impact of a limited extension of influenza vaccine offer from a decision maker perspective, who have to counterbalance limited resources with good public health strategies. Furthermore, age-based immunization strategies have proved to be more feasible and efficacious than those targeting at-risk subjects.^57–60^

These advantages are counterbalanced by the major investments needed to reach the set targets. However, stratifying the 50–64-year age-class into three equally distributed groups allowed us to investigate which policies would yield the greatest benefits and to better evaluate the impact of progressively reducing the recommended age of immunization.

We estimated cost savings from the restricted perspective of third-party payers; estimates made on the basis of a more comprehensive societal perspective would presumably yield further savings. Indeed, a previous study estimated substantial opportunity costs in terms of lost productivity due to ILIs.^50^

The strength of our study is that it used real-world input data to estimate subjects at risk of influenza and its complications and influenza cases requiring/not requiring ED access. Specifically, community-acquired ILI cases were estimated through the active epidemiological surveillance network of sentinel GPs coordinated by the Istituto Superiore di Sanità of the Ministry of Health. Cases of ILI and LRTI cases requiring ED access were estimated through the syndromic surveillance system that monitors ILI daily on the basis of ED accesses in the referral hospitals for adults in Liguria. This system displays high sensitivity in capturing suspected cases, as the chief complaints recorded by ED admission software are scanned for keywords suggestive of ILI and LRTI syndromes, and data folders are automatically reviewed. It also offers high specificity once each case captured has been critically reviewed according to case definitions. In order to take into account the natural variability of the attack rate, we calculated the mean incidence rates of ILI/LRTI over the last six seasons by age-group.

Although influenza vaccines confer protection only against influenza virus infection, our model is conservatively based on ILIs and LRTIs, rather than laboratory-confirmed influenza. This is justified by the high resources needed to ascertain the specific incidence of influenza at the community level. Furthermore, the main clinical impact of influenza is due to bacterial superinfections.^50,61^ In addition, we integrated SSS data with data from the recently implemented Chronic Condition Data Warehouse (CCDWH). This records data gathered from multiple Medicare data sources (hospital discharge records, drug consumption and expenditure, medical fee exemptions, outpatient visits and laboratory/imaging procedures) within a specific period, thereby enabling the main risk factors and their distribution by age-group to be predicted.

To our knowledge, this is the first static model investigating the impact of lowering the recommended age for influenza immunization that has considered the exclusive use of QIV in adults aged 50–64 years. In addition, our study included vaccine administration costs among the costs of influenza immunization and estimated the costs of the entire healthcare pathway, making the analysis more realistic and complete.

The study has some limitations, mainly due to the limited data on some parameters. To overcome this problem, we adjusted the available data on adults residing in the GMA, for example in our estimate of national ILI incidence rates among WRF and NRF subjects aged 50–64 years from aggregate surveillance data. Furthermore, evaluation of the epidemiological burden could be improved by including influenza-related death among the outcomes considered and taking into account the impact of illness on health-related quality of life. The type of model used constitutes another limitation; being static, it could not capture some epidemiological aspects such as herd immunity. Finally, no sensitivity analysis was performed on the results.

In summary, the results suggest that lowering the age recommendation for influenza vaccination would allow a higher proportion of at-risk subjects to be reached and reduce the societal, financial and healthcare burden of influenza and its complications. The proposed policies applied easily attainable VC objectives and adopted a pragmatic strategy targeting restricted age-groups.

Moreover, decision-makers could decide to implement these policies in different steps and choice the more suitable starting scenario in base of advantages and drawbacks of each approach, according to the evidence-based assessment on how to best allocate limited resources.

## Supplementary Material

Supplemental Material
