# Supplementary material for: Lowering the recommended age for the free and active offer of influenza vaccination in Italy: clinical and economic impact analysis in the Liguria region
Source: Hum Vaccin Immunother. 2020 Oct 29;17(5):1387–95. doi: 10.1080/21645515.2020.1810494 (PMC8078656; doi:10.1080/21645515.2020.1810494)
Supplement: Supplemental Material [file KHVI_A_1810494_SM5775.docx]

**Supplementary Table 2. Cost estimates broken down by hypothesized scenario, age-group and risk factor**

| **Variables (€)** | **Age-group (years)** | **Hypothetical 1** | | | **Difference (hypothetical 1 vs base case)** | **Hypothetical 2** | | | **Difference (hypothetical 2 vs base case)** | **Hypothetical 3** | | | **Difference (hypothetical 3 vs base case)** | **Hypothetical 4** | | | **Difference (hypothetical 4 vs base case)** |
| --- | --- | --- | --- | --- | --- | --- | --- | --- | --- | --- | --- | --- | --- | --- | --- | --- | --- |
|  |  | **At least one risk factor** | **No risk factor** | **Total** |  | **At least one risk factor** | **No risk factor** | **Total** |  | **At least one risk factor** | **No risk factor** | **Total** |  | **At least one risk factor** | **No risk factor** | **Total** |  |
| Vaccination cost | 50-54 | 90,047 | 58,655 | 148,702 | 63,077 | 63,031 | 146,644 | 209,675 | 124,050 | 90,047 | 146,644 | 236,691 | 87,989 | 63,031 | 58,655 | 121,686 | 36,061 |
|  | 55-59 | 99,376 | 49,668 | 149,043 | 64,193 | 69,562 | 124,166 | 193,728 | 108,878 | 99,376 | 124,166 | 223,542 | 74,498 | 69,562 | 49,668 | 119,230 | 34,379 |
|  | 60-64 | 112,236 | 38,287 | 150,523 | 66,089 | 78,568 | 95,717 | 174,284 | 89,850 | 112,236 | 95,717 | 207,953 | 57,430 | 78,568 | 38,287 | 116,854 | 32,420 |
|  | Total | 301,658 | 146,610 | 448,268 | 193,358 | 211,161 | 366,527 | 577,688 | 322,778 | 301,658 | 366,527 | 668,185 | 413,276 | 211,161 | 146,610 | 357,770 | 102,861 |
| Flu treatment, | 50-54 | 49,229 | 126,204 | 175,433 | -13,856 | 56,149 | 113,307 | 169,456 | -19,833 | 49,229 | 113,307 | 162,536 | -12,897 | 56,149 | 126,204 | 182,352 | -6,937 |
|  | 55-59 | 54,313 | 115,905 | 170,218 | -14,716 | 61,954 | 104,059 | 166,013 | -18,921 | 54,313 | 104,059 | 158,372 | -11,846 | 61,954 | 115,905 | 177,859 | -7,076 |
|  | 60-64 | 74,599 | 86,047 | 160,647 | -18,375 | 85,084 | 77,251 | 162,335 | -16,687 | 74,599 | 77,251 | 151,850 | -8,797 | 85,084 | 86,047 | 171,131 | -7,890 |
|  | Total | 178,142 | 328,155 | 506,297 | -46,947 | 203,187 | 294,616 | 497,803 | -55,441 | 178,142 | 294,616 | 472,758 | -80,487 | 203,187 | 328,155 | 531,342 | -21,902 |
| *Treatment, in ED* | 50-54 | 30,336 | 41,618 | 71,954 | -7,661 | 34,603 | 37,367 | 71,970 | -7,645 | 30,336 | 37,367 | 67,703 | -4,251 | 34,603 | 41,618 | 76,220 | -3,395 |
|  | 55-59 | 33,463 | 44,286 | 77,749 | -8,411 | 38,177 | 39,761 | 77,938 | -8,222 | 33,463 | 39,761 | 73,224 | -4,525 | 38,177 | 44,286 | 82,463 | -3,698 |
|  | 60-64 | 51,052 | 30,839 | 81,892 | -11,881 | 58,229 | 27,685 | 85,914 | -7,859 | 51,052 | 27,685 | 78,737 | -3,155 | 58,229 | 30,839 | 89,068 | -4,704 |
|  | Total | 114,852 | 116,742 | 231,594 | -27,953 | 131,009 | 104,812 | 235,821 | -23,726 | 114,852 | 104,812 | 219,664 | -39,884 | 131,009 | 116,742 | 247,751 | -11,796 |
| Complications | 50-54 | 152,968 | 225,428 | 378,395 | -38,971 | 174,473 | 202,395 | 376,868 | -40,498 | 152,968 | 202,395 | 355,363 | -23,033 | 174,473 | 225,428 | 399,901 | -17,466 |
|  | 55-59 | 205,237 | 235,000 | 440,237 | -50,651 | 234,123 | 210,986 | 445,108 | -45,779 | 205,237 | 210,986 | 416,222 | -24,014 | 234,123 | 235,000 | 469,122 | -21,765 |
|  | 60-64 | 406,642 | 218,383 | 625,025 | -93,992 | 463,800 | 196,052 | 659,852 | -59,165 | 406,642 | 196,052 | 602,694 | -22,331 | 463,800 | 218,383 | 682,183 | -36,834 |
|  | Total | 764,846 | 678,811 | 1,443,657 | -183,614 | 872,396 | 609,433 | 1,481,829 | -145,442 | 764,846 | 609,433 | 1,374,279 | -252,991 | 872,396 | 678,811 | 1,551,206 | -76,064 |
| *Complications requiring hospitalization* | 50-54 | 98,198 | 88,396 | 186,593 | -23,657 | 112,009 | 79,367 | 191,376 | -18,874 | 98,198 | 79,367 | 177,565 | -9,029 | 112,009 | 88,396 | 200,405 | -9,846 |
|  | 55-59 | 144,794 | 118,973 | 263,767 | -34,616 | 165,189 | 106,817 | 272,005 | -26,377 | 144,794 | 106,817 | 251,610 | -12,156 | 165,189 | 118,973 | 284,161 | -14,221 |
|  | 60-64 | 338,375 | 128,941 | 467,316 | -76,902 | 385,943 | 115,751 | 501,694 | -42,524 | 338,375 | 115,751 | 454,126 | -13,190 | 385,943 | 128,941 | 514,884 | -29,334 |
|  | Total | 581,366 | 336,310 | 917,676 | -135,175 | 663,141 | 301,935 | 965,076 | -87,775 | 581,366 | 301,935 | 883,301 | -169,549 | 663,141 | 336,310 | 999,450 | -53,400 |
